# P-197. Summary of Recurrent *Clostridioides difficile* Infections using Whole Genome Sequencing, Minnesota 2019-2021

**DOI:** 10.1093/ofid/ofae631.401

**Published:** 2025-01-29

**Authors:** Bree E Friedman, Daniel R Evans, Kelly Pung, Bonnie Weber, Xiong Wang, Christine Lees, Stacy Holzbauer

**Affiliations:** University of Minnesota, St. Paul, Minnesota; Minnesota Department of Health, St Paul, Minnesota; Minnesota Department of Health, St Paul, Minnesota; Minnesota Department of Health, St Paul, Minnesota; Minnesota Department of Health, St Paul, Minnesota; Minnesota Department of Heatlh, St. Paul, Minnesota; Minnesota Department of Health, St Paul, Minnesota

## Abstract

**Background:**

Recurrent *Clostridioides difficile* infection (CDI) is typically defined as a positive *C. difficile* toxin or assay in the 8 weeks following an incident CDI. However, positive detections outside of this timeframe could represent failure to clear the initial infection and positive detections inside this timeframe could represent a new CDI based on molecular analysis of *C. difficile* isolates. We performed whole genome sequencing on consecutive isolates of patients to compare and summarize isolate pairs in Minnesota.

**Methods:**

We analyzed data from our active population- and laboratory-based surveillance for CDI as part of CDC’s Emerging Infections Program from 2019-2021. An incident CDI was defined as the first positive *C. difficile* toxin or molecular assay on a stool specimen from a person at least 1 year of age during the study period. A recurrent CDI was defined as an additional positive test > 2 weeks following an incident infection. Stool specimens were sent to the Minnesota Department of Health for *C. difficile* culture. DNA was extracted using QIAmp BiOstic Bacteremia DNA Kit, sequencing libraries prepared using Illumina’s DNA Prep Kit, and sequencing performed using Illumina MiSeq and NextSeq platform.

**Results:**

There were 252 patients with two consecutive *C. difficile* isolates cultured and sequenced. Ninety-three (37%) of second instance CDI isolates had a sequence type (ST) that did not match the ST of their previous CDI, indicating a new infection and not a relapse of initial ST. Some (23%) relapses occurred outside the standard 8-week timeframe (range 58-486 days). The time between infections was significantly shorter for relapses (median 33 vs 50 days, p< 0.0001). Older age (≥ 65, p< 0.0001), antibiotic use prior to incident CDI (p=0.0003), and incident CDI that were hospital-onset or following recent hospitalization (p=0.0014) were associated with relapse.

**Conclusion:**

Over a third of the patients with recurrent CDIs did not have a relapse of their initial ST. For those with a relapse, almost a quarter occurred greater than 8 weeks from their initial infection. While time between CDI episodes is important in determining new infection or relapse, there are other factors to consider such as age, antibiotic history, and hospital associated infections.

**Disclosures:**

**All Authors**: No reported disclosures

